# Supplementary material for: Label-free structural imaging of plant roots and microbes using third-harmonic generation microscopy
Source: Sci Rep. 2025 Oct 16;15:36186. doi: 10.1038/s41598-025-20030-9 (PMC12533127; doi:10.1038/s41598-025-20030-9)
Supplement: Supplementary file 5 — Supplementary Information [file 41598_2025_20030_MOESM5_ESM.pdf]

Label-free structural imaging of plant roots and microbes using third-harmonic generation microscopy: Supplementary Information

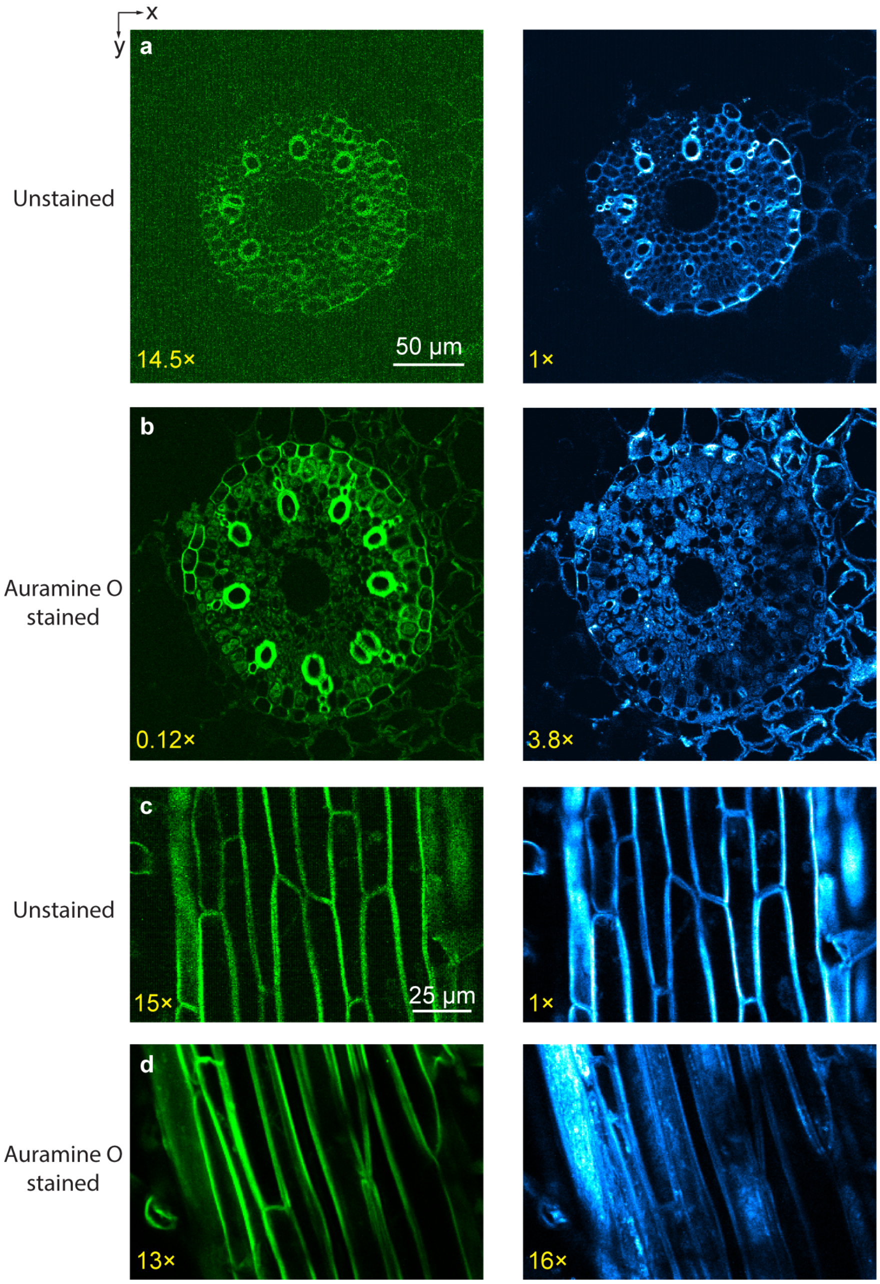

Supplementary Figure S1: Fluorescence staining disrupts the inner structures of root samples. (**a,b**) 3PF (green) and THG (cyan) xy images of transverse root sections of mature zones. (**c,d**) 3PF (green) and THG (cyan) xy images of lateral sections of mature zones from two root samples. Roots are (**a,c**) unstained or (**b,d**) stained with Auramine O to label lignin-rich structures, including cell walls. All images were acquired

at 0.5 µm/pixel. The brightness of both 3PF and THG channels in (**a,b**) and (**c,d**) was normalized to their respective THG images of unstained roots, with digital gain values listed at the bottom left of each image. (Larger gains indicate dimmer images.) Clearly, staining reduces brightness in the THG channels in (**b,d**), indicative of structural disruption and decreased optical inhomogeneity within the cells caused by staining. Post-objective power: (**a**) 3.4 mW, (**b**) 1.7 mW, (**c**) 1.9 mW, (**d**) 4.2 mW.

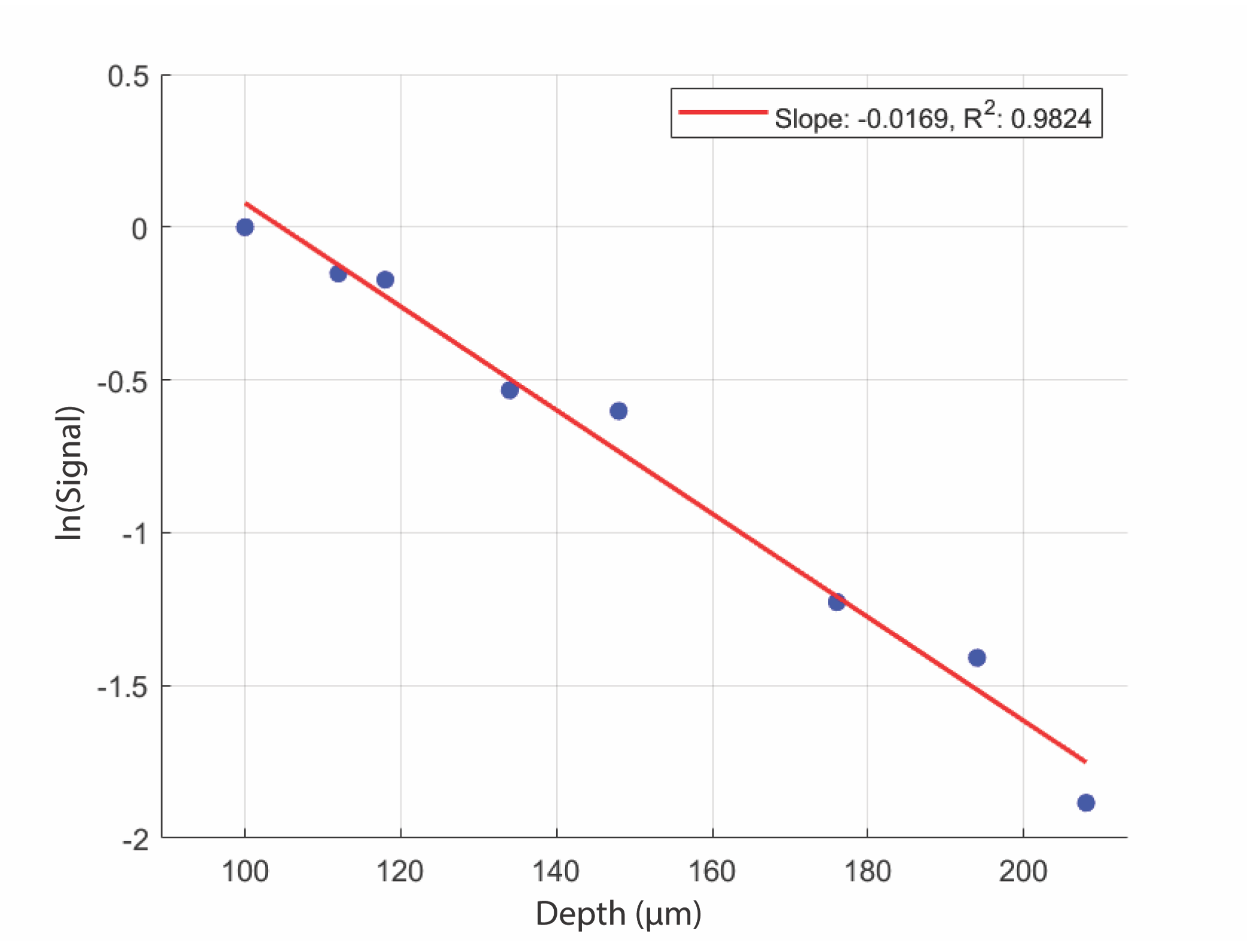

Supplementary Figure S2: Semi-logarithmic plot of THG signal attenuation with depth in the meristem region of *B. distachyon* root, calculated using the image stack shown in Figure 4. The natural logarithm of the THG signal from bright striated features was measured at 8 different depths. As THG signal decays with depth z according to $\exp(-3z/\mathrm{EAL})$, linear fitting of $\ln(\mathrm{Signal})$ indicates an effective attenuation length (EAL) of 177 µm at a 1300 nm excitation wavelength.
